# Hypoxia-Inducible Factor-2α Promotes Liver Fibrosis by Inducing Hepatocellular Death

**DOI:** 10.3390/ijms252313114

**Published:** 2024-12-06

**Authors:** Raja Gopal Reddy Mooli, Dhanunjay Mukhi, Mikayla Watt, Veerababu Nagati, Sara M. Reed, Nikita K. Gandhi, Michael Oertel, Sadeesh K. Ramakrishnan

**Affiliations:** 1 Division of Endocrinology and Metabolism, Department of Medicine, University of Pittsburgh, Pittsburgh, PA 15261, USA; dhanunjay.mukhi@pennmedicine.upenn.edu (D.M.); mwatt@unc.edu (M.W.); veerababu.nagati@utrgv.edu (V.N.); smr187@pitt.edu (S.M.R.); nkg13@pitt.edu (N.K.G.); 2 Pittsburgh Liver Research Center, University of Pittsburgh, Pittsburgh, PA 15261, USA; mio19@pitt.edu; 3 Division of Experimental Pathology, Department of Pathology, University of Pittsburgh, Pittsburgh, PA 15261, USA; 4 McGowan Institute for Regenerative Medicine, University of Pittsburgh, Pittsburgh, PA 15261, USA; 5 Hillman Cancer Center, University of Pittsburgh, Pittsburgh, PA 15261, USA

**Keywords:** hepatocyte, hypoxia, hypoxia-inducible factor-2α, fibrosis, apoptosis

## Abstract

The activation of hypoxia-inducible factors (HIF)-1α and 2α in the liver is closely linked to the progression of fatty liver diseases. Prior studies indicated that disrupting hepatocyte HIF-2α attenuates diet-induced hepatic steatosis, subsequently decreasing fibrosis. However, the direct role of hepatocyte HIF-2α in liver fibrosis has not been addressed. Hepatic HIF-2α expression was examined in mouse model of carbon tetrachloride (CCl_4_)-induced liver fibrosis. Conditional hepatocyte *Hif-2α* knockout mice were employed to investigate the role of hepatocyte HIF-2α in fibrosis. Markers of apoptosis, proliferation, inflammation, and fibrosis were assessed through biochemical, molecular, and histological analyses. We found an induction of HIF-2α in CCL_4_-injected liver injury and fibrosis mouse models. Hepatocyte-specific deletion of HIF-2α attenuated stellate cell activation and fibrosis, with no significant difference in inflammation. Disrupting hepatocyte HIF-2α led to reduced injury-mediated hepatocellular apoptosis. Surviving hepatocytes exhibited hypertrophy, which was strongly associated with the activation of c-JUN signaling. Our study demonstrates a direct role of hepatocyte HIF-2α in liver fibrosis by promoting hepatocyte apoptosis. The reduction in apoptosis and induction of hepatocyte hypertrophy following HIF-2α disruption is closely linked to enhanced c-JUN signaling, a survival mechanism in response to liver injury. These findings highlight HIF-2α as a potential therapeutic target for liver fibrosis.

## 1. Introduction

Liver fibrosis can progress to cirrhosis and is responsible for up to 40% of deaths in developed countries [1,2]. It is characterized by the excessive accumulation of the extracellular matrix (ECM), primarily crosslinked collagens of type I and type III, which disrupts liver structure and function [2,3]. Fibrosis is initiated by the activation of hepatic stellate cells (HSCs) and portal fibroblasts, leading to the proliferation of ECM-expressing myofibroblasts [3,4]. HSCs are also activated by mitogens, such as platelet-derived growth factor and transforming growth factor-β (TGF-β) [3,5]. Additionally, inflammatory immune cells, predominantly macrophages, are the major source of TGF-β, playing a critical role in HSC activation and fibrosis [6,7,8]. Moreover, hepatocytes are the primary cells affected during liver injury, leading to changes in their gene and secretion profiles, which can promote fibrosis [9,10]. For example, the expression of hepatocyte-enriched transcription factors and genes was repressed in injured liver, and they release intracellular molecules known as damage-associated molecular patterns (DAMPs) that trigger fibrosis [11,12]. Excessive wound healing due to uncontrolled hepatocyte death can result in fibrogenesis [13]. On the other hand, persistent liver injury triggers the compensatory proliferation of surviving hepatocytes, potentially leading to carcinogenesis [14]. The factors that influence hepatocyte survival mechanisms during injury and fibrosis remain unclear, highlighting the need to understand these mechanisms to identify novel targets for liver fibrosis.

Hypoxia signaling, mediated by the transcription factors hypoxia-inducible factor (HIF)-1α and HIF-2α [15], plays a crucial role in liver injury [16,17]. HIFs are heterodimeric proteins composed of an oxygen-sensitive subunit (HIF-α) and an oxygen-insensitive subunit (HIF-β). Under normoxia, prolyl hydroxylase domain (PHD) enzymes hydroxylate HIF-α at specific proline residues using oxygen as a substrate. Hydroxylated HIF is then ubiquitinated by the Von Hippel Lindau (VHL) complex, an E3 ubiquitin ligase, and is then subsequently degraded in the proteasome [18,19]. HIF-α is also regulated by oxygen-independent mechanisms, such as tricarboxylic acid (TCA) metabolites, inflammatory cytokines, and reactive oxygen species (ROS) [20,21]. Elevated levels of HIF-1α and HIF-2α are associated with liver dysfunction in both alcoholic and metabolic dysfunction-associated steatotic liver diseases [22]. Mouse models of bile duct ligation and carbon tetrachloride (CCl_4_) combined with moderate alcohol feeding show that HIF-1α promotes liver fibrosis [22,23]. However, patients with hepatitis and cholestasis, express similar levels of HIF-1α and HIF-2α [24]. Genetic models with HIF-2α overexpression in hepatocytes show a significant dysregulation of lipid homeostasis leading to spontaneous steatosis and hypercholesterolemia, culminating in liver fibrosis [25,26]. Consistently, deletion of HIF-2α in hepatocytes attenuates diet-induced hepatic steatosis and fibrosis [27]. However, it is difficult to discern whether the attenuation of liver fibrosis is secondary to lower steatosis or a direct effect of hepatocyte HIF-2α [27]. Lentiviral-mediated knockdown of HIF-2α in the liver attenuated CCl_4_-induced liver fibrosis [24]. However, lentiviral vectors non-specifically target all cell types, including hepatocytes. Thus, the causal role of hepatocyte HIF-2α in liver fibrosis remains elusive.

In this study, we investigated the role of hepatocyte HIF-2α in liver fibrosis using a CCl_4_-induced liver injury mouse model. Temporal deletion of HIF-2α in hepatocytes reduced the expression of fibrotic markers independent of inflammation. Hepatocyte-specific deletion of HIF-2α attenuated hepatocyte death and resulted in hepatocyte hypertrophy and was strongly associated with elevated levels of c-Jun, a pro-survival signaling mechanism crucial for hepatocyte survival and liver regeneration.

## 2. Results

### 2.1. HIF-2α Expression Is Upregulated in Acute Liver Injury and Fibrosis

**Figure 1:**
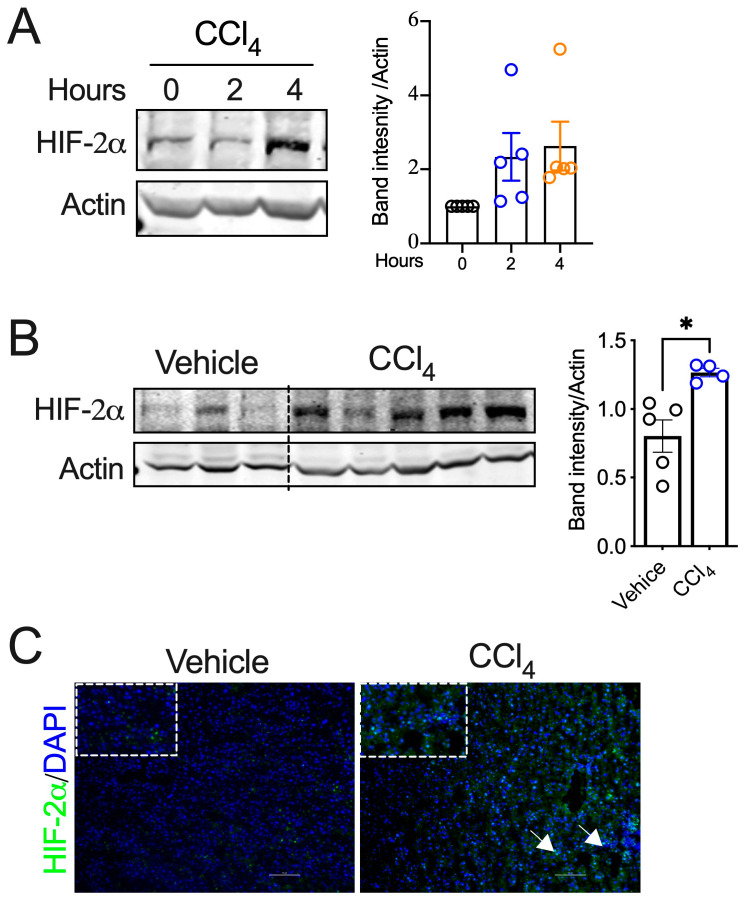
HIF-2α is activated in liver injury and fibrosis models. (**A**) Western blot and band intensity quantification graph showing HIF-2α in liver lysates at 0 h, 2 h, and 4 h after CCl_4_ injection in C57BL6 mice. (**B**) Western blot analysis of HIF-2α expression in liver lysates from the C57BL6 mice. (**C**) Immunofluorescence analysis of HIF-2α expression in livers from C57BL6 mice treated with CCl_4_. Images were acquired using a Nikon Eclipse fluorescence microscope at 20X magnification. Scale bars indicate 100 μm length. *n* = 5–6 mice per group. White arrows indicating HIF-2α expression. All the data are presented as mean ± SEM. *p* < 0.05 (*), as analyzed by a two-tailed Student’s *t*-test.

Acute liver injury induces hypoxia signaling mediated by HIF-1α and HIF-2α [28,29]. Our analysis showed that liver injury induced the expression of HIF-2α as early as 4 h post-injury (Figure 1A,B). Using immunostaining, we show that HIF-2α stabilization occurred in cells with prominent nuclei, which are likely hepatocytes (Figure 1C). These data indicate that HIF-2α is upregulated in liver injury, raising the possibility that it may play a role in liver fibrosis.

### 2.2. Hepatocyte-Specific HIF-2α Inhibition Protects Against Liver Fibrosis

**Figure 2:**
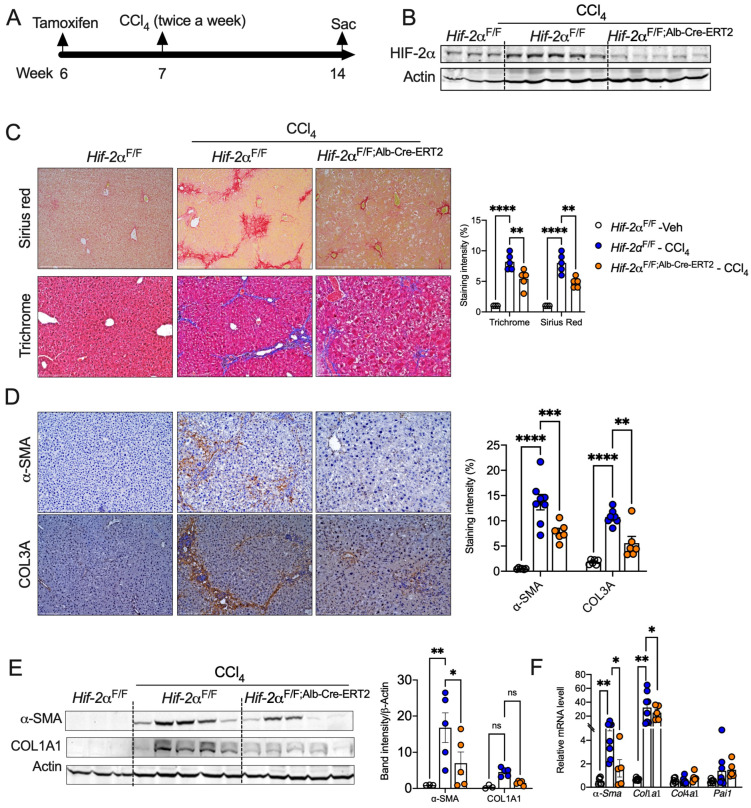
Hepatocyte-specific disruption of HIF-2α attenuates stellate cell activation and fibrosis. (**A**) Schematic showing corn oil or CCl_4_ (0.6 mL/kg body weight) injection to *Hif-2α*^F/F^ and *Hif-2α*^F/F;Alb-Cre-ERT2^ mice. (**B**) Western blot analysis of HIF-2α in the liver lysates from corn oil (*Hif-2α*^F/F^) or CCl_4_-injected *Hif-2α*^F/F^ mice and knockout *Hif-2α*^F/F;Alb-Cre-ERT2^ mice. (**C**) Sirius red and trichrome staining and intensity quantification in liver of *Hif-2α*^F/F^ and *Hif-2α*^F/F;Alb-Cre-ERT2^ mice injected with CCl_4_. (**D**) IHC analysis of α-SMA and COL3A and staining quantification in CCl_4_-injected *Hif-2α*^F/F^ and *Hif-2α*^F/F;Alb-Cre-ERT2^ mice. Images were acquired at 20X magnification, and scale bars indicate 200 μm length. (**E**) Western blotting analysis of α-SMA and COl1A1. *n* = 6 mice per group. (**F**) mRNA expression levels for the indicated genes in CCl_4_-injected *Hif-2α*^F/F^ and *Hif-2α*^F/F;Alb-Cre-ERT2^ mice. Experiments performed in two cohorts with both males and females. ns = non-significant. All the data are presented as mean ± SEM. *p* < 0.05 (*); or *p* <0.005 (**); or *p* < 0.001 (***) or *p* < 0.0001 (****), as analyzed by one-way ANOVA (Tukey multiple comparisons test).

To test our hypothesis that HIF-2α induction in hepatocytes promotes fibrosis independent of steatosis, we used mice with temporal disruption of HIF-2α, specifically in hepatocytes (*Hif-2α*^F/F;Alb-Cre-ERT2^) [30]. One week after tamoxifen treatment, mice were administered CCl_4_ twice a week for 7 weeks (Figure 2A). As expected, HIF-2a expression was strongly induced in CCl_4_-treated *Hif-2α*^F/F^ mice but not in *Hif-2α*^F/F;Alb-Cre-ERT2^ mice (Figure 2B). Histological analysis using Sirius red and Masson’s trichrome staining showed a significant reduction in liver fibrosis in *Hif-2α*^F/F;Alb-Cre-ERT2^ mice compared to *Hif-2α*^F/F^ mice (Figure 2C). Similarly, *Hif-2α*^F/F;Alb-Cre-ERT2^ mice showed reduced lipid accumulation and fibrosis in a diet-induced fibrosis model (Supplementary Figure S1).

HSCs transdifferentiate into myofibroblast-like cells, which rapidly proliferate and secrete numerous pro-fibrotic molecules around the area of injury [31,32]. Among these molecules, alpha-smooth muscle actin (α-SMA) serves as a marker of HSC activation [25]. Immunohistochemical analysis showed increased expression of α-SMA in the livers of *Hif-2α*^F/F^ mice, whereas its expression was significantly downregulated in *Hif-2α*^F/F;Alb-Cre-ERT2^ mice (Figure 2D). Additionally, collagen type III A (COL3A) expression was elevated in the livers of *Hif-2α*^F/F^ mice but not in *Hif-2α*^F/F;Alb-Cre-ERT2^ mice (Figure 2D). Similarly, Western blot analysis showed increased protein levels for α-SMA and collagen type 1A (COL1A) in the livers of *Hif-2α*^F/F^ mice but not in *Hif-2α*^F/F;Alb-Cre-ERT2^ mice (Figure 2E). Furthermore, mRNA levels of *α-Sma* were significantly reduced in CCl_4_-treated *Hif-2α*^F/F;Alb-Cre-ERT2^ mice (Figure 2F). However, no differences were observed in the mRNA levels of *Col1a1*, *Col4a1*, and plasminogen activator inhibitor-1 (*Pai-1*) between *Hif-2α*^F/F^ and *Hif-2α*^F/F;Alb-Cre-ERT2^ mice (Figure 2F). To further investigate whether HIF-2α in HSCs modulates fibrosis, we treated the HSC cell line LX2 with TGFβ in the presence or absence of hypoxia. An increase in HIF-2α in hypoxia-exposed LX2 cells did not modulate TGFβ-mediated induction of COL1A1 at the protein level (Supplementary Figure S2). Thus, our data reveal that hepatocyte HIF-2α regulates hepatic stellate cell activation and fibrosis.

### 2.3. HIF-2α Induces Fibrosis Independent of Inflammation

**Figure 3:**
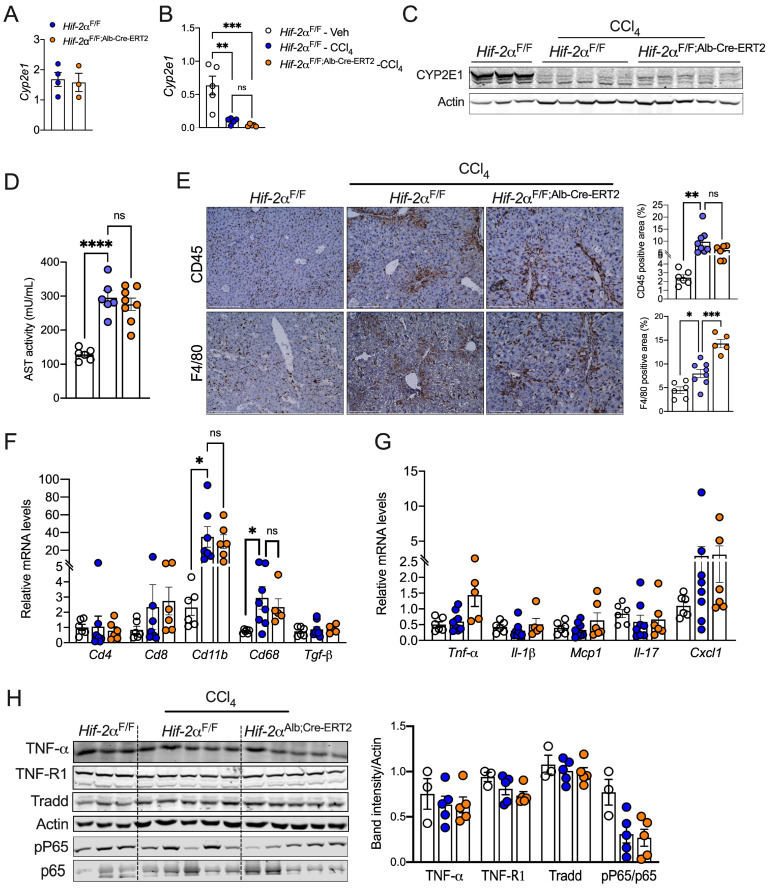
Hepatocyte HIF-2α does not affect injury-mediated liver inflammation. Quantitative PCR analysis of *Cyp2e1* expression in *Hif-2α*^F/F^ and *Hif-2α*^F/F;Alb-Cre-ERT2^ mice fed on (**A**) standard chow diet and (**B**) CCl_4_-treated mice. (**C**) Western blotting analysis of CYP2E1 expression in livers of CCl_4_-treated mice. (**D**) Serum AST levels in CCl_4_-injected *Hif-2α*^F/F^ and *Hif-2α*^F/F;Alb-Cre-ERT2^ mice. (**E**) Immunostaining for CD45 and F4/80 proteins in the livers from CCl_4_-injected *Hif-2α*^F/F^ and *Hif-2α*^F/F;Alb-Cre-ERT2^ mice. Images were acquired at 20X magnification and scale bars indicate 200 μm length. *n* = 6 mice per group. (**F**,**G**) Quantitative PCR analysis of cytokines and chemokines. (**H**) Western blot showing proteins involved in inflammatory signaling in CCl_4_-injected *Hif-2α*^F/F^ and *Hif-2α*^F/F;Alb-Cre-ERT2^ mice. ns = non-significant. All the data are presented as mean ± SEM. *p* < 0.05 (*); or *p* < 0.005 (**); or *p* < 0.001 (***); or *p* < 0.0001 (****), as analyzed by one-way ANOVA (Tukey multiple comparisons test).

Next, we sought to determine how the disruption of hepatocyte HIF-2α attenuates liver fibrosis. The enzyme cytochrome P450 2E1 (CYP2E1) metabolizes CCl_4_, and *Cyp2e1* KO mice are resistant to CCl_4_-induced hepatotoxicity [33]. Given that hypoxia represses cytochrome P450 activity and expression [34], we assessed whether HIF-2α regulates liver fibrosis by modulating xenobiotic metabolism. We observed no difference in *Cyp2e* expression between *Hif-2α*^F/F^ and *Hif-2α*^F/F;Alb-Cre-ERT2^ mice assessed under steady-state conditions (Figure 3A). Moreover, CCl_4_ treatment reduced the mRNA and protein levels of CYP2E1 in both *Hif-2α*^F/F^ and *Hif-2α*^F/F;Alb-Cre-ERT2^ mice (Figure 3B,C), suggesting that HIF-2α may not alter CCl_4_ metabolism. Additionally, we observed no difference in serum aspartate transaminase (AST) levels, indicating that the decreased fibrosis is not due to improved liver damage (Figure 3D).

Inflammatory cells are critical drivers of liver fibrosis [35]. We assessed whether reduced fibrosis in *Hif-2α*^F/F;Alb-Cre-ERT2^ mice is driven by attenuated inflammatory response. Immunostaining revealed no difference in the number of CD45+ cells between *Hif-2α*^F/F^ and *Hif-2α*^F/F;Alb-Cre-ERT2^ mice (Figure 3E). Paradoxically, F4/80+ cells were higher in *Hif-2α*^F/F;Alb-Cre-ERT2^ mice, indicating an increase in macrophages (Figure 3E). qPCR analysis showed an increase in the mRNA levels of *Tnf-α* in *Hif-2α*^F/F;Alb-Cre-ERT2^ mice; however, the expression of other cytokines and macrophage markers was not different between the two groups (Figure 3F,G). Activation of inflammatory pathways, such as NF-kB and TNF-R1, induces a number of pro-fibrotic factors [36,37]. However, we did not observe any changes between the genotypes (Figure 3H). Together, the data suggest that attenuated fibrosis observed in the *Hif-2α*^F/F;Alb-Cre-ERT2^ mice is not due to a decrease in hepatic inflammation.

### 2.4. HIF-2α Deletion Prevents Hepatocyte Apoptosis in Fibrotic Livers

**Figure 4:**
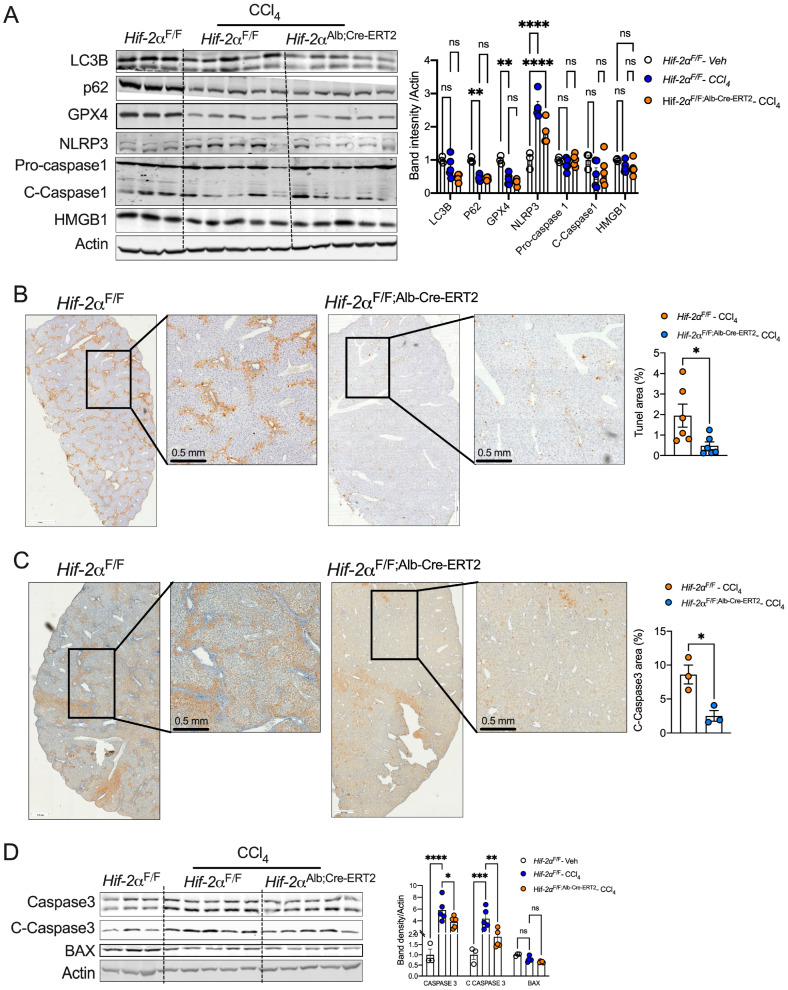
Hepatocyte-specific disruption of HIF-2α attenuates cell death (**A**) Western blot showing markers for autophagy, ferroptosis, and pyroptosis of livers from CCl_4_-injected *Hif-2α*^F/F^ and *Hif-2α*^F/F;Alb-Cre-ERT2^ mice. (**B**,**C**) TUNEL staining and immunostaining for cleaved caspase 3 (C-Caspase 3) in liver samples from CCl_4_-treated *Hif-2α*^F/F^ and *Hif-2α*^F/F;Alb-Cre-ERT2^ mice. Images were acquired using a PreciPoint microscope at 20X magnification. (**D**) Western blot showing proteins for apoptosis markers. ns = non-significant. All the data are presented as mean ± SEM. *p* < 0.05 (*); or *p* < 0.005 (**); or *p* < 0.001 (***) or *p* < 0.0001 (****), analyzed by one-way ANOVA (Tukey multiple comparisons test) and a two-tailed Student’s *t*-test.

Apoptosis of injured hepatocytes significantly contributes to liver fibrosis [38]. Moreover, necroptosis, pyroptosis, ferroptosis, and autophagy are dysregulated in injury-mediated liver fibrosis and NASH [39,40,41,42]. In our study, hepatocyte-specific deletion of HIF-2α did not affect markers of pyroptosis (NLRP3, cleaved caspase-1), autophagy (p62 and LC3 II/I), and ferroptosis (GPX4) (Figure 4A). To investigate whether HIF-2α influences hepatocyte cell death, we performed terminal deoxynucleotidyl transferase dUTP nick-end labeling (TUNEL) staining. Our results showed increased TUNEL-positive cells in CCl_4_-treated fibrotic livers from *Hif-2α*^F/F^ mice, indicating apoptotic cell death. However, the number of apoptotic cells was significantly reduced in *Hif-2α*^F/F;Alb-Cre-ERT2^ mice (Figure 4B). Immunohistochemistry for cleaved caspase 3 revealed a similar increase in apoptosis in *Hif-2α*^F/F^ mice but not in *Hif-2α*^F/F;Alb-Cre-ERT2^ mice (Figure 4C). Additionally, Western blot analysis showed decreased protein levels of total caspase 3 and cleaved caspase 3 in *Hif-2α*^F/F;Alb-Cre-ERT2^ mice compared to *Hif-2α*^F/F^ mice, but no change in BAX expression was observed (Figure 4D). These results suggest that hepatocyte HIF-2a promotes hepatocyte apoptosis.

### 2.5. Disruption of HIF-2α Enhances Hepatocyte Survival and c-JUN Activation

**Figure 5:**
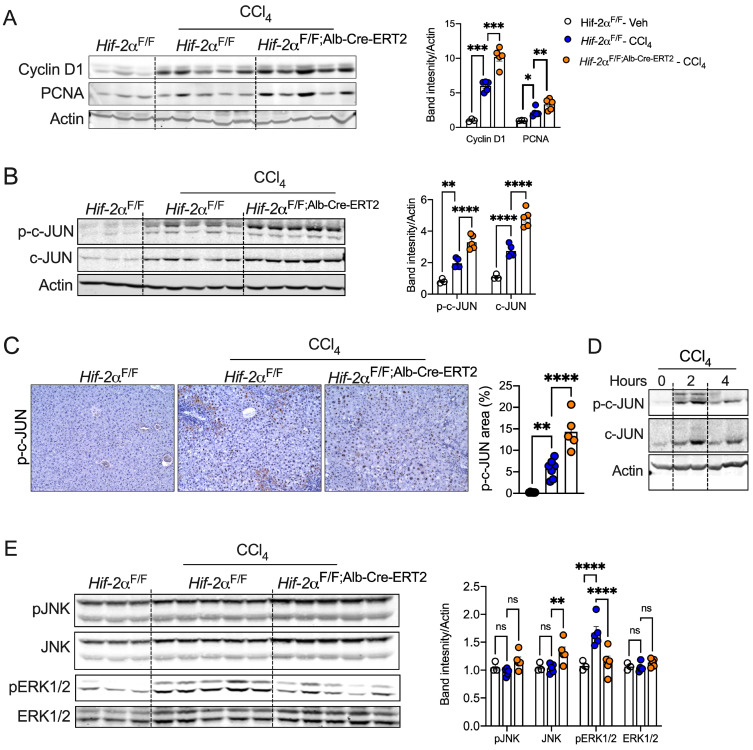
Hepatocyte HIF-2α deletion activates cell survival mechanisms in response to injury. (**A**) Western blotting analysis for PCNA and cyclin D1 of livers from CCl_4_-injected *Hif-2α*^F/F^ and *Hif-2α*^F/F;Alb-Cre-ERT2^ mice. (**B**) Western blot showing c-JUN and p-c-JUN in the livers of chronic CCl_4_ treatment of *Hif-2α*^F/F^ and *Hif-2α*^F/F;Alb-Cre-ERT2^ mice. (**C**) Immunohistochemical analysis of p-c-JUN in *Hif-2α*^F/F^ and *Hif-2α*^F/F;Alb-Cre-ERT2^ mice. Images were acquired at 20X magnification and scale bars indicate 200 μm length. *n* = 6 mice per group. (**D**) Western blot showing c-JUN and p-c-JUN in liver lysates at 2 h and 4 h after CCl_4_ injection in C57BL6 mice. (**E**) Western blot showing c-JUN regulatory signaling in the livers of *Hif-2α*^F/F^ and *Hif-2α*^F/F;Alb-Cre-ERT2^ mice. ns = non-significant. All the data are presented as mean ± SEM. *p* < 0.05 (*); or *p* < 0.005 (**) or *p* < 0.001 (***); or *p* < 0.0001 (****), as analyzed by one-way ANOVA (Tukey multiple comparisons test).

Hepatocyte survival and proliferation are transcriptionally regulated, protecting against injury-mediated fibrosis and inflammation [43,44]. We observed a significant increase in the protein levels of cyclin D1 and PCNA in CCl_4_-treated *Hif-2α*^F/F;Alb-Cre-ERT2^ mice compared to *Hif-2α*^F/F^ mice (Figure 5A). Cyclin D1 and PCNA expression are regulated by the transcription factor c-Jun, which is crucial for the survival of injured hepatocytes [45,46,47,48]. Western blot analysis revealed increased expression of c-JUN and phosphorylated c-JUN protein levels in chronic CCl_4_-treated *Hif-2α*^F/F;Alb-Cre-ERT2^ mice compared to *Hif-2α*^F/F^ mice (Figure 5B). c-JUN elicits discordant effects on liver injury depending on the cell type. For instance, activation of c-JUN in hepatic stellate cells or myofibroblasts induces fibrosis, whereas, in hepatocytes, c-JUN promotes survival and regeneration [48,49,50,51,52]. Immunostaining revealed intense phospho-c-JUN staining in the hepatocytes of CCl_4_-treated *Hif-2α*^F/F;Alb-Cre-ERT2^ mice (Figure 5C). In addition to chronic CCl_4_ treatment models, acute treatment also elevated c-JUN phosphorylation in *Hif-2α*^F/F;Alb-Cre-ERT2^ mice compared to *Hif-2α*^F/F^ mice (Figure 5D). c-JUN expression and activation are modulated by upstream kinases, such as c-JUN N-terminal kinase (JNK) and extracellular regulated kinase (ERK1/2) [45]. We observed a modest decrease in ERK1/2 phosphorylation and a trend towards an increase in JNK levels in *Hif-2α*^F/F;Alb-Cre-ERT2^ mice (Figure 5E). Together, these data demonstrate that the hepatocyte HIF-2α regulates c-JUN phosphorylation in liver injury, and the induction of hepatocyte c-JUN in mice with disruption of hepatocyte HIF-2α could be associated with protection against CCl_4_-induced liver fibrosis.

### 2.6. HIF-2α Inhibition Promotes Hepatocyte Expansion and Preserves Cell Identity

**Figure 6:**
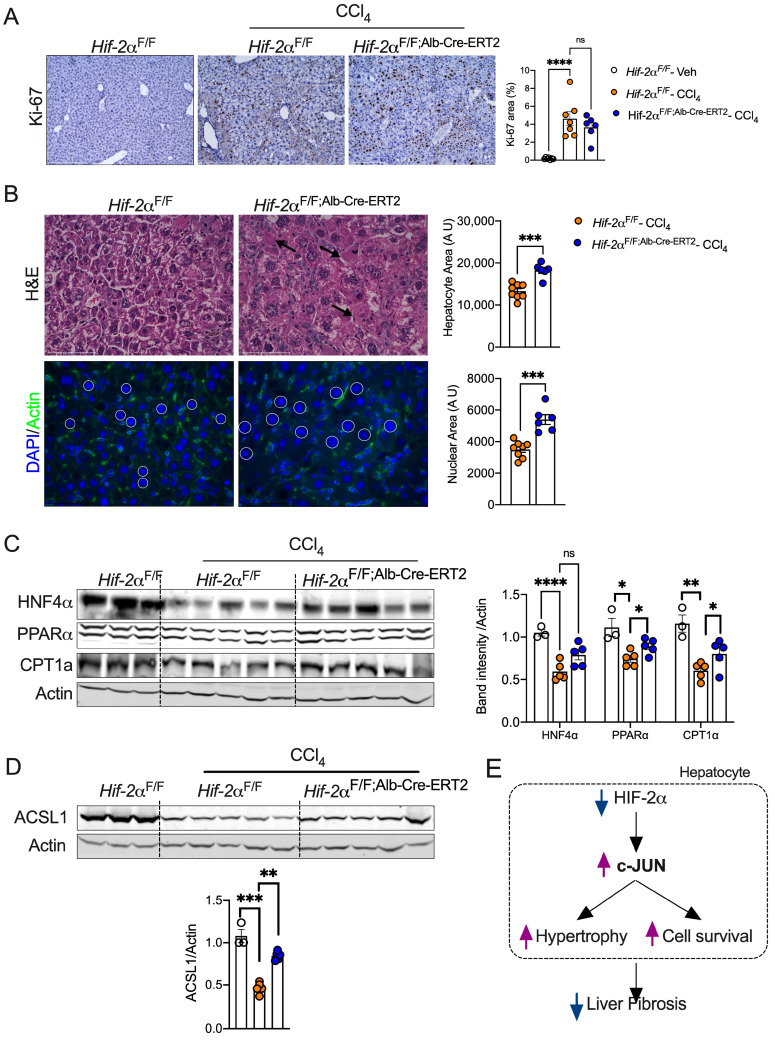
HIF-2α inhibition preserved hepatocyte cell identity and expansion. (**A**) Ki-67 immunostaining in liver from mice administered with CCl_4_. Images were acquired at 20X magnification and scale bars indicate 200 μm length. (**B**) H&E and immunofluorescence for actin/DAPI staining images of livers from CCl_4_-injected *Hif-2α*^F/F^ and *Hif-2α*^F/F;Alb-Cre-ERT2^ mice were acquired at 60X, and the scale bar indicates 75 μm. Nuclei were encircled by a white line. (**C**) Western blotting analysis of *Hif-2α*^F/F^ and *Hif-2α*^F/F;Alb-Cre-ERT2^ mice injected with CCl_4_. (**D**) Western blot for ACSL1. (**E**) Graphical abstract showing the mechanisms by which HIF-2α attenuates fibrosis. ns = non-significant. All the data are presented as mean ± SEM. *p* < 0.05 (*); or *p* < 0.005 (**); or *p* < 0.001 (***); or *p* < 0.0001 (****), as analyzed by one-way ANOVA (Tukey multiple comparisons test) and a two-tailed Student’s *t*-test.

We next examined whether hepatocyte HIF-2α regulates cell proliferation in liver injury and found no difference in Ki-67-positive cells in CCl_4_-treated *Hif-2α*^F/F;Alb-Cre-ERT2^ mice (Figure 6A). Chronic CCl_4_ treatment increased the nuclear and hepatocyte size in *Hif-2α*^F/F;Alb-Cre-ERT2^ mice, indicating hepatocyte hypertrophy (Figure 6B). Hepatocyte-enriched transcription factors, such as hepatocyte nuclear factor (HNF4α), play a critical role in hepatocellular identity and protect against injury-mediated dysfunction and fibrosis [53]. We found a significant increase in HNF4α in *Hif-2α*^F/F;Alb-Cre-ERT2^ mice (Figure 6C). Moreover, the expression of lipid metabolism genes, such as PPARα, CPT1α, and ACSL1, whose repression is linked to injury-mediated hepatocyte death [54], recuperates in CCl_4_-treated *Hif-2α*^F/F;Alb-Cre-ERT2^ mice (Figure 6C,D). Collectively, our data suggest that disruption of HIF-2α in hepatocytes induces adaptive hypertrophy and preserves hepatocyte identity in the liver injury model.

## 3. Discussion

Hypoxia, prevalent in liver injury, induces HIF-1α and HIF-2α, which are known to contribute to the expression of pro-fibrotic genes [32,55,56,57,58]. The role of HIF-1α in liver fibrosis is well-documented [22,56,59]. This study elucidates the cell-autonomous function of hepatocyte HIF-2α in liver fibrosis using a CCl_4_-induced liver injury model. We show that both acute and chronic liver injuries induce HIF-2α in hepatocytes. Deletion of HIF-2α specifically in hepatocytes attenuates liver fibrosis, partly due to a reduction in hepatocyte death. Furthermore, disruption of HIF-2α promotes hepatocyte hypertrophy, an adaptive survival mechanism against cellular injury. Finally, our data indicate that the disruption of hepatocyte HIF-2α enhances injury-induced activation of c-JUN, a key transcription factor involved in hepatocyte survival, and preserves the markers of hepatocellular identity. Together, these findings demonstrate that hepatocyte HIF-2α acts as a critical driver of liver fibrosis by promoting hepatocyte death, associated with the attenuation of injury-mediated induction of c-JUN (Figure 6E).

Several interventions targeting hypoxic signaling have shown promise in preserving liver function during inflammation and fibrosis. For instance, overexpression of VHL, an E3 ubiquitin ligase that targets HIF-α for proteasomal degradation, prevents liver injury [24]. Similarly, loss of prolyl hydroxylase domain (PHD) enzymes attenuates liver fibrosis in a bile duct injury model [60]. Several studies have shown that HIF-2α is activated in various liver diseases, including steatosis, NASH, and hepatocellular carcinoma models [27,61,62], which are interconnected with each other [28]. Additionally, HIF-2α inhibition protects against fibrosis in NASH models, potentially through improved lipotoxicity, ROS, and inflammation [25,63]. For example, liver injury induces reactive oxygen species and induces NFκB signaling, which triggers and mediates NLRP3 inflammasomes’ activation to produce pyroptotic death of hepatocytes via caspase-1 [64,65]. Inhibition of HIF-2α also modulates immune cells, such as macrophages, which are key players in fibrosis [66]. Given the complex role of HIF-2α in hepatic steatosis and inflammation, its causal role in fibrosis remained unclear. Our data suggest that HIF-2α promotes liver fibrosis through hepatocyte-intrinsic mechanisms independent of steatosis and inflammation.

Hepatocyte apoptosis is a common and critical component in liver injury, including in patients with NASH, alcoholic hepatitis, and chemically-induced fibrosis [67,68]. Apoptosis of damaged hepatocytes drives fibrosis by activating liver myofibroblasts, making the regulation of hepatocyte apoptosis crucial for liver homeostasis [68,69]. Attenuating hepatocyte apoptosis and increasing their survival can ameliorate fibrosis through various mechanisms. Recent studies have shown that hepatocyte apoptotic bodies can activate hepatic stellate cells [67]. For example, mice lacking apoptotic caspases are protected against liver injury and fibrosis [41,70]. Interestingly, inhibition of apoptosis using caspase inhibitors has been shown to reduce liver injury and fibrosis [71,72]. Thus, all these studies provide evidence that protecting hepatocytes from apoptosis is sufficient to attenuate liver injury and fibrosis. Moreover, we found that HIF-2α regulates cleaved caspase and hepatocyte apoptosis in liver injury, which is consistent with previous reports identifying HIF-2α as an inducer of apoptosis [62,73,74]. Therefore, we anticipate that HIF-2α promotes fibrosis by regulating hepatocyte survival mechanisms in liver injury.

Activation of hepatocyte survival transcription factors in response to liver injury has been shown to ameliorate liver fibrosis by regulating various signaling pathways. In particular, c-JUN is an important regulator of hepatocyte survival by regulating hepatocyte proliferation in various injury-mediated liver pathologies [48,75]. For example, c-JUN promotes hepatocyte survival in hepatitis and chemically induced stress models [76]. A study by Schulien et.al has shown that hepatocyte specific deletion of c-JUN mice is more prone to liver fibrosis [47]. In addition, it was shown that cyclin D1 that plays a critical role in hepatocyte survival, which is the downstream target of c-JUN [45]. Moreover, it has been also shown that while HIF-1α induces c-JUN expression, HIF-2α inhibits its expression [77]. Our study shows abundant levels of c-JUN and its phosphorylated form in CCl_4_-induced liver fibrosis, which were further enhanced by the deletion of HIF-2α in hepatocytes. Additionally, our data showed an increase in the expression of cyclin D1 in response to injury under deletion of HIF-2α in hepatocytes. Furthermore, the induction of c-JUN in CCl_4_-treated *Hif-2α*^F/F;Alb-Cre-ERT2^ mice is associated with hepatocyte hypertrophy, a survival mechanism that inhibits apoptotic cell death. Thus, we propose that HIF-2α deletion attenuates liver fibrosis partly by inhibiting c-JUN-mediated hepatocyte survival and hypertrophy.

Hepatocyte nuclear factors (HNFs), including HNF1, HNF3, HNF4, and other hepatocyte-enriched transcriptional networks, such as FOXA2, C/EBPα, PPARα, and CPT1α, control hepatocyte differentiation and function [10,12,43,78]. For example, constitutive expression of HNF4α attenuates fibrosis by protecting hepatocytes against apoptosis and inhibiting epithelial-mesenchymal transition [79]. Moreover, resetting the hepatocyte-specific transcriptional network, particularly HNF4α, preserves hepatocyte function and inhibits liver fibrosis [12]. Thus, reestablishing dysregulated hepatocyte-specific transcription factors protects injured hepatocytes from chronic liver injury. Inhibition of HIF-2α increases the expression of hepatic genes, such as HNF4α, ACSL1, PPARα, and CPT1α [61,80]. We observed that disrupting hepatocyte HIF-2α increases the expression of HNF4α, ACSL1, and CPT1α in CCl_4_-treated mice, suggesting that hepatocyte HIF-2α inhibits hepatocyte-enriched genes and transcriptional machinery. Therefore, targeting the HIF-2α-HNF4α axis may provide potential therapeutic target for liver fibrosis.

In conclusion, we propose that HIF-2α in hepatocytes promotes injury-mediated liver fibrosis in a cell-autonomous manner. HIF-2α drives fibrosis by enhancing apoptosis that is strongly associated with c-JUN signaling, a survival mechanism. Additionally, markers of functional hepatocytes are enhanced in hepatocytes with disrupted HIF-2α. These findings provide a strong rationale for further exploring the therapeutic potential of HIF-2α inhibitors for treating liver fibrosis.

### Limitations of the Study

Apoptotic bodies derived from hepatocytes activate the stellate cells and induce fibrosis [41,67]. Consistently, apoptosis inhibitors reduce liver injury and fibrosis [71,72] indicating that hepatocyte apoptosis could promote the progression of liver disease. We show that HIF-2a regulates hepatocyte survival mechanisms and fibrosis. Our future studies will understand the mechanistic underpinnings of HIF-2α-mediated hepatocyte survival in regulating liver fibrosis

## 4. Materials and Methods

### 4.1. Animals

Conditional *Hif-2α* knockout mice *(Hif-2α*^F/F;Alb-Cre-ERT2^) were generated by crossing *Hif-2α*^F/F^ mice with mice expressing tamoxifen-inducible *Cre* recombinase driven by the albumin promoter (Alb-Cre-ERT2). Littermates (*Hif-2α*^F/F^) that did not express *Cre* recombinase were used as controls. Animals were maintained under a 12 h day and night cycle with free access to food and water throughout the experiment. For chronic treatment, 6–8 week-old male *Hif-2α*^F/F;Alb-Cre-ERT2^ and *Hif-2α*^F/F^ mice were injected intraperitoneally with tamoxifen (Cayman Chemicals, Ann Arbor, MI, USA) AT (200 mg/kg bodyweight) in corn oil for three consecutive days. Twenty-four hours before carbon tetrachloride (CCl_4_) injection, animals were moved into a biosafety cabinet for acclimatization. For the fibrosis models, mice were injected intraperitoneally with CCl_4_ in corn oil (0.6 mL/kg body weight) twice a week for 7 weeks. Animals were sacrificed three days after the last CCl_4_ injection. For acute treatment, 8-week-old C57BL6 mice was administered intraperitoneally with a single dosage of CCl_4_ (1 mL/kg body weight) and the mice were sacrificed at different time points. Control mice received corn oil intraperitoneally throughout the experiments. For diet-induced fibrosis model, 6–8 week-old male *Hif-2α*^F/F;Alb-Cre-ERT2^ and *Hif-2α*^F/F^ mice were injected intraperitoneally with tamoxifen, and 1 week later, the mice were provided with a CDAA-HFD with 60% kcal% fat with 0.1% methionine and no added choline (AO6071302, Research Diets Inc., New Brunswick, NJ, USA) for the final 8 weeks of the study. Animals handling procedures followed and approved by the Institutional Animal Care and Use Committee (IACUC) at the University of Pittsburgh (IACUC Approval # 24044761).

### 4.2. Cell Culture

The human hepatic stellate cell line (LX2) obtained from the Department of Pathology at the University of Pittsburgh was cultured in DMEM supplemented with 10% fetal bovine serum with 1% penicillin–streptomycin at 37 C in 5% CO_2_. After 75–80% confluence in 12-well plates, the cells were treated with TGF-β (MedChem Express, Monmouth Junction, NJ, USA), after exposure to 1% hypoxia. The detailed information about the treatment duration and dosage were described in the figure legends.

### 4.3. RNA Isolation, cDNA Synthesis, and qPCR Analysis

Total RNA from the liver was extracted using Trizol reagent (Invitrogen, Carlsbad, CA, USA) following manufacturer’s instructions [81]. Here, 1 μg of total RNA was reverse transcribed (cDNA) using M-MLV reverse transcriptase (Invitrogen) for 5 min at 25 °C (annealing), 120 min at 42 °C (cDNA synthesis), and 5 min at 95 °C (enzyme denaturation). Gene expression at mRNA levels was analyzed by comparative CT (2^−CT^) method with Radiant SYBR green PCR mix (#QY9001, Alkali Scientific Inc., Fort Lauderdale, FL, USA) on a QuantStudio-3 instrument (#A28137, Applied Biosystems, Carlsbad, CA, USA). The relative expression of each gene was represented after normalizing with β-actin. Primers sequences were listed in Supplementary Table S1.

### 4.4. Western Blotting

Liver lysates were prepared in radio immunoprecipitation assay lysis buffer (RIPA, 50 mM Tris-HCl, 150 mM NaCl, 2 mM EDTA, 1% NP-40, and 0.1% SDS, pH 7.5) supplemented with protease and phosphatase inhibitors. About 25 μg of protein was subjected to polyacrylamide gel electrophoresis at 100 V and transferred onto a nitrocellulose membrane. Membranes were blocked with 3% skim milk in Tris-buffered saline (pH 7.5) and probed with primary antibodies overnight at 4 °C. They were then incubated with IR-conjugated secondary antibodies (Cell Signaling Technology, Inc., Danvers, MA, USA) for one hour at room temperature. Images were visualized using an Odyssey CLx Imaging System (LI-COR, Inc., Lincoln, NE, USA) and quantified using ImageJ Software Version 1.5g (NIH, Bethesda, MD, USA). Antibodies and their source are listed in Supplementary Table S2.

### 4.5. H&E, Sirius Red, and Masson’s Trichrome Staining

Liver tissues were fixed overnight in 10% phosphate-buffered saline, embedded in paraffin, and sectioned (5 microns). The liver sections were stained with hematoxylin and eosin (H&E) as per the standard protocol. For Sirius Red staining, slides were incubated with Picrosirius Red stain (#24901-250, Polyscience Inc., Warrington, PA, USA) for one hour until equilibrium was achieved, then washed under tap water and rinsed in PBS several times. For Masson’s Trichrome staining, slides were incubated in Bouin’s fixative for one hour at room temperature, and we followed the manufacturer’s protocol (#ab150686, Abcam, Waltham, MA, USA).

#### Immunohistochemistry Staining

FFPE sections were deparaffinized and antigen-retrieved using citrate buffer (10 mM Na_3_C_6_H_5_O_7_ and 0.05% Tween 20, pH 6.0). Sections were blocked with 5% goat serum for 30 min and incubated with primary antibodies (αSMA, COL3A, CD45, F4/80, p-c-JUN, and Ki-67) overnight at 4 °C. After washing with PBS-Tween 20 (PBST), sections were incubated with secondary antibodies conjugated with horseradish peroxidase (HRP) (#SK-4100, Vector labs, Newark, CA, USA) for 30 min at room temperature. Sections were developed using 3,3′-diaminobenzidnie (DAB) and counterstained with hematoxylin. Antibodies and their source are listed in Supplementary Table S2. For determining apoptosis, terminal deoxynucleotidyl transferase-mediated dUTP nick-end labelling (TUNEL) was performed on FFPE sections as described.

### 4.6. Immunofluorescence Staining

OCT-embedded frozen livers sections (5 microns) were fixed with 4% ice-cold paraformaldehyde for 10 min and permeabilized with 0.1% Triton X-100 in PBS. Sections were blocked with 5% goat serum in PBS for 30 min at room temperature. Slides were washed with PBST and incubated overnight at 4 °C with anti-HIF-2α and anti-actin antibodies (1:100 dilution). Sections were then incubated with Alexa flour-conjugated secondary antibodies (#A11034, Invitrogen, Carlsbad, CA, USA) for one hour at room temperature, washed with PBST, and mounted with anti-fading mounting medium containing DAPI (#P36935; Invitrogen, Carlsbad, CA, USA). Antibodies and their source are listed in Supplementary Table S2.

### 4.7. Microscopy, Imaging, and Staining Quantification

Stained tissue sections were mounted using Permount toluene solution (immunohistochemistry) or DAPI (immunofluorescence). All the images of stained sections were captured using an EVOS-Auto2 fluorescence microscope (Invitrogen, Carlsbad, CA, USA) or an AxioObserver Z1 microscope (Carl Zeiss, Carl-Zeiss-Str, Oberkochen, Germany). Hepatocyte and nuclear size were quantified using ImageJ software with approximately 10 hepatocytes measured per animal, and the average size per mouse was presented in a bar graph. All the IHC staining quantifications were determined using ImageJ software and considered 4–6 mice per group with 3–4 images per mice.

### 4.8. Statistical Analysis

Statistical significance was calculated using GraphPad prism 9.0 software (Version 9.0.0). Results were expressed as mean ± SEM. Significant differences among the groups were calculated using *t*-tests (two groups) and one-way ANOVA (three groups). A *p*-value <0.05 was considered significant. The staining intensity for immunohistochemistry and band intensity for Western blotting were quantified using ImageJ software.

## Data Availability

The data presented in this study are available from the corresponding author upon reasonable request.

## Supplementary Materials

The following supporting information can be downloaded at: https://www.mdpi.com/article/10.3390/ijms252313114/s1.
