# Supplementary material for: Hypoxia-Inducible Factor-2α Promotes Liver Fibrosis by Inducing Hepatocellular Death
Source: Int J Mol Sci. 2024 Dec 6;25(23):13114. doi: 10.3390/ijms252313114 (PMC11642083; doi:10.3390/ijms252313114)
Supplement: Supplementary file 1 [file ijms-25-13114-s001.zip › Supplementary data.pdf]

Supplementary Table S1. List of Primers

Gene	Forward primer (‘5-3’)	Reverse primer (‘5-3’)
Hif-2α	AGCTTCCTTCGGACACATAAG	GCTTTCAGGTACAAGTTATCCATTT
Hif-1α	GGGTACAAGAAACCACCCAT	GAGGCTGTGTCGACTGAGAA
α-Sma	GTTCAGTGGTGCCTCTGTCA	ACTGGGACGACATGGAAAAG
Col1a1	TAGGCCATTGTGTATGCAGC	ACATGTTCAGCTTTGTGGACC
Col4a1	CACATTTTCCACAGCCAGAG	GTCTGGCTTCTGCTGCTCTT
Pai-1	ACGCCTGGTGCTGGTGAATGC	ACGGTGCTGCCATCAGACTTGTG
Tgf-β	CAACCCAGGTCCTTCCTAAA	GGAGAGCCCTGGATACCAAC
CD11b	ATGGACGCTGATGGCAATACC	TCCCCATTCACGTCTCCCA
CD68	ACTTCGGGCCATGTTTCTCT	GCTGGTAGGTTGATTGTCGT
Tnf-α	AGGGTCTGGGCCATAGAACT	CCACCACGCTCTTCTGTCTAC
Mcp-1	AGCTCTCTCTTCCTCCACCA	GGCGTTAACTGCATCTGGCT
CD4	TCCTTCCCACTCAACTTTGC	AAGCGAGACCTGGGGTATCT
CD8	GCTCAGTCATCAGCAACTCG	ATCACAGGCGAAGTCCAATC
Cxcl1	CTGCACCCAAACCGAAGTC	AGCTTCAGGGTCAAGGCAAG
Il-17A	TCTCCACCGCAATGAAGACC	GACCAGGATCTCTTGCTGGA
Il-1β	CCAAGCAACGACAAAATACC	GTTGAAGACAAACCGTTTTTCC
Cyp2e1	TTCCCTAAGTATCCTCCGTGA	CGTAATCGAAGCGTTTGTTG
β-actin	TATTGGCAACGAGCGGTTCC	GGCATAGAGGTCTTTACGGATGT

Supplementary Table S2. List of primary antibodies

S.No	Antibody	Catalog	Manufacturer
1	HIF-2α	NB100-122	Novus
2	Collagen3A	22734-1-AP	Protientech
3	Collagen1	14695-1-AP	Protientech
4	α-SMA	19245S	Cell Signaling Technology
5	CD45	70257S	Cell Signaling Technology
6	F4/80	70076S	Cell Signaling Technology
7	p-c-Jun	3270S	Cell Signaling Technology
8	c-Jun	9165S	Cell Signaling Technology
9	p-JNK	9255L	Cell Signaling Technology
10	JNK	3708S	Cell Signaling Technology
11	pERK	4370S	Cell Signaling Technology
12	ERK	4696S	Cell Signaling Technology
13	Cyp2E1	HPA009128	Sigma
14	CyclinD1	2978T	Cell Signaling Technology
15	CyclinE1	11554-1-AP	Proteintech
16	c-Caspase 3	9661	Cell Signaling Technology
17	Bax	50599-2-Ig	Proteintech
18	Actin	66009-1-Ig	Proteintech
19	ACSL1	4047	Cell Signaling Technology
20	HNF4α	Sc-374229	Santa Cruz
21	CPT1a	PA5-29995	Invitrogen
22	Caspase 3	9662	Cell Signaling Technology
23	NLRP3	15101	Cell Signaling Technology
24	GPX4	52455S	Cell Signaling Technology
25	HMGB1	ab18256	Abcam
26	LC3A/B	12741S	Cell Signaling Technology
27	C caspase 1	89332	Cell Signaling Technology
