# Supplementary material for: Hypoxia-Inducible Factor-2α Promotes Liver Fibrosis by Inducing Hepatocellular Death
Source: Int J Mol Sci. 2024 Dec 6;25(23):13114. doi: 10.3390/ijms252313114 (PMC11642083; doi:10.3390/ijms252313114)
Supplement: Supplementary file 1 [file ijms-25-13114-s001.zip › Supplementary figure Data.pdf]

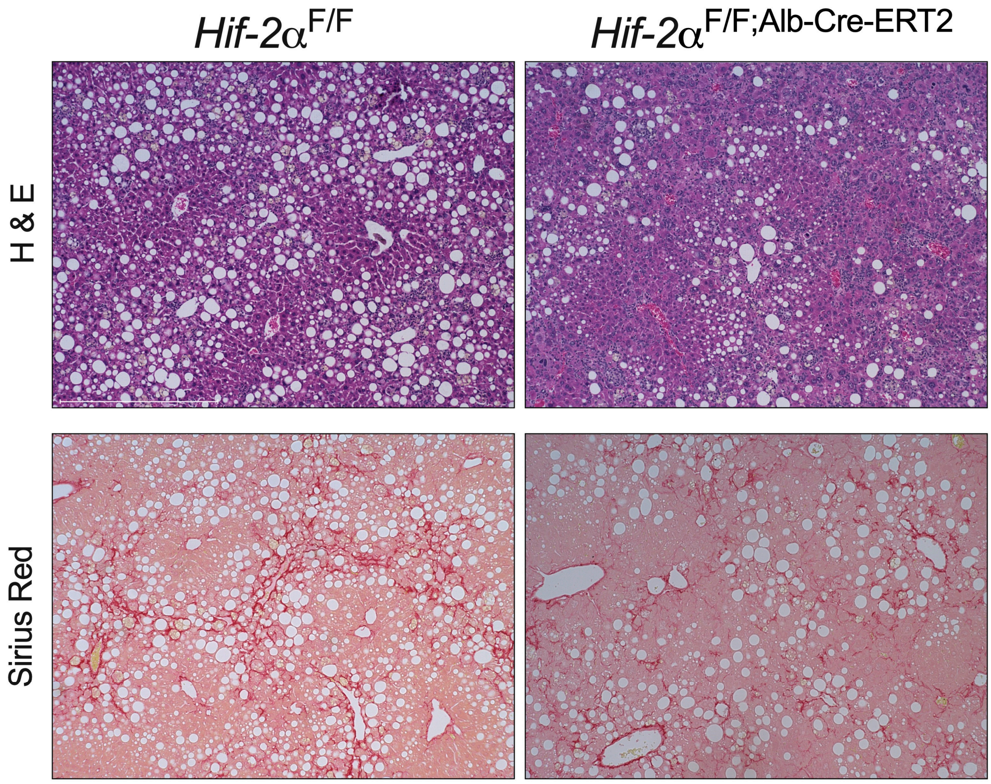

Figure S1: HIF-2α deletion protects from diet-induced fibrosis. H&E and Sirius Red staining of in liver of Hif-2αF/F and Hif-2αF/F;Alb-Cre-ERT2 mice provided with CDAA- HFD for 8-weeks.

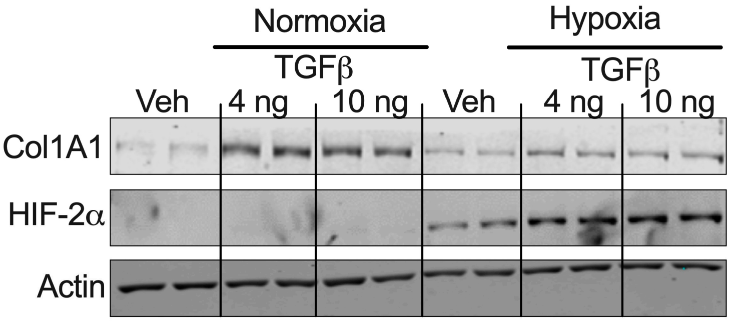

Figure S2: HIF-2α in hepatic stellate cells does not regulate fibrosis. LX-2 cells exposed to normoxia and hypoxia for 16 hours in the presence or absence of TGF-b.
